# Enuresis and overactive bladder in children: what is the relationship between these two conditions?

**DOI:** 10.1590/S1677-5538.IBJU.2015.0579

**Published:** 2016

**Authors:** Ariane Sampaio Sousa, Maria Luisa Veiga, Ana Aparecida N. Braga, Maria Clara Carvalho, Ubirajara Barroso

**Affiliations:** 1 Escola Bahia de Medicina e Saúde Pública – Pos – Graduação, Salvador, Brasil; 2 Departamento Urologia, Universidade Federal da Bahia (UFBA), Salvador, Bahia, Brasil

**Keywords:** Urinary Bladder, Enuresis, Child, Urinary Incontinence

## Abstract

**Objective:**

Evaluate clinical aspects associated with the presence of nocturnal enuresis (NE) in children with a diagnosis of overactive bladder (OAB).

**Material and Methods:**

A data base of 200 children who were evaluated by a structured questionnaire was analysed retrospectively . OAB was defined as the presence of urinary urgency (n=183 cases) and/or daytime urinary incontinence associated with holding maneuvers (n=168 cases). Inclusion criteria were a confirmed diagnosis of OAB, age 5-16 years, and no anatomical or neurological alterations of the urinary tract. Patients were divided into enuretics and non-enuretics. The two groups were compared with respect to sex, age, skin color, presence urinary infection, urgency, urge incontinence, non-urge incontinence, pollakiuria, urinary dysfunction, nocturia, holding maneuvers, number of episodes of enuresis and bowel alterations. In a univariate analysis, the chi-square test was used to compare proportions, with p-values <0.05 being considered significant. A multivariate analysis was conducted to identify independent predictive factors.

**Results:**

Enuresis was diagnosed in 141/200 children. The two groups were similar with respect to sex, age and skin color. No difference was found in relation to urinary infection, non-urge incontinence, urinary dysfunction, nocturia, encopresis or constipation. The two groups were significantly different with regard to some symptoms related to OAB such as urgency (p=0.001), urge incontinency (p=0.001) and holding maneuvers (p=0.033). Following multivariate analysis, only holding maneuvers (p=0.022) remained as an independent predictive factor.

**Conclusion:**

The only independent predictive factor for resolution of enuresis in children with OAB, as detected in the multivariate analysis, was holding maneuvers.

## INTRODUCTION

The International Children's Continence Society classifies enuresis as monosymptomatic (MSE) when bedwetting is the only symptom and non-monosymptomatic (non-MSE) when lower urinary tract symptoms (LUTS) are present (1). Enuresis is a common condition in children, affecting around 15-20% of 5-year olds, 5-10% of 7-year olds, 5% of 10-year olds and 1-3% of children of 15 years of age (2–4). Older studies have reported daytime LUTS in around 15-40% of cases of enuresis (5, 6). However, more recent data suggest that LUTS such as daytime incontinence, urgency, frequency and voiding postponement are present in 50-80% of cases (3, 7). Daytime incontinence has been found more often in girls than in boys, whereas enuresis is more common in boys (8). In many cases, LUTS go unrecognized in patients with enuresis because doctors fail to ask about them or because parents are far more concerned about bedwetting (9, 10). Because enuresis tends to have a greater effect on family dynamics than LUTS, it may be more perceptible to parents.

Differentiating between the types of enuresis (MSE and non-MSE) is relevant because the physiopathology and management may not be the same for the two conditions. Non-MSE is more commonly associated with urinary tract infections (UTI), vesicoureteral reflux, constipation and behavioral problems (11, 12). Parents often believe that their child's treatment has failed entirely when enuresis persists even though complete resolution of LUTS was achieved. Since recognizing each condition individually is important, patients with enuresis should be investigated for LUTS and patients with LUTS should be asked about the presence of enuresis. Although OAB is commonly associated with enuresis, the clinical relationship between these two conditions remains to be clarified. In fact, identifying the characteristics of these patients is a crucial step towards gaining a better picture of the clinical scenario, prognosis and management of both conditions. Therefore, the objective of the present study was to evaluate the clinical aspects associated with enuresis in children and adolescents with OAB.

## MATERIAL AND METHODS

A database of 200 children who were evaluated by a structured questionnaire was analyzed retrospectively. OAB was defined as the presence of urgency (n=183 cases) and/or daytime incontinence (n=168 cases). The inclusion criteria were a confirmed diagnosis of OAB, age between 5 and 16 years and the absence of any anatomical alterations or neurogenic disorders of the lower urinary tract. Patients for whom the database information was incomplete, and those who had recorded fewer than 4 voids per day in the bladder diary were excluded from the study. The internal review board of the Escola Bahiana de Medicina e Saúde Pública approved the study protocol under reference number 12141113.0.0000.5544.

A structured questionnaire was administered to all the patients to obtain the following information: demographic data (age, sex and skin color), number of voids, the presence of nocturnal enuresis (and its intensity), daytime incontinence (and its intensity), nocturia, urgency, straining, holding maneuvers, constipation (in accordance with the Rome III criteria), and a history of UTI confirmed by culture. Daytime incontinence was classified as urge or non-urge incontinence. Daytime incontinence and enuresis were classified according to intensity as daily, three times a week or more, less than three times a week or occasional.

Holding maneuvers were considered present when the child's parents reported typical body posturing such as squeezing the genitals, crossing the legs, going on tiptoe or squatting on the heel (Vincent's curtsy). All patients underwent uroflowmetry plus electromyography. Dysfunctional voiding was defined as an abnormal voiding pattern in the uroflow curve and activity on electromyography (flat curve plus activity on electromyography, staccato and interrupted voiding). Only 85 patients completed a 3-day bladder diary. The association between enuresis and the number of voids, the presence of daytime incontinence, and constipation was evaluated. Patients with and without enuresis were compared with respect to the presence of all the aforementioned clinical variables.

The SPSS software program, version 20.0 was used throughout analysis. In a univariate analysis, the chi-square test was used to compare proportions, with p-values <0.05 being considered statistically significant. Multivariate analysis was conducted for identifying the independent predictor variables. The variables that were significant in the univariate analysis were consecutively added to a multivariate hierarchical model, which started with a simple predictor.

## RESULTS

Of the 200 children with a diagnosis of OAB, 84 were boys (42%) and 116 girls (58%). Age ranged from 5 to 16 years, with a mean of 8.6±2.9 years. NE was diagnosed in 141 children (70.5%). Of these children, 83 (58.9%) were female. The mean age of the children with enuresis was 8.6±2.7 years, while the mean age of those without enuresis was 8.6±3.2 years (p=0.831). In 112 patients (80.6%), NE occurred at least three times a week. NE rate of 68% (n=87) in children ≤9 years and 75% (n=54) in those 10 years of age or older.

Regarding the demographic characteristics of the enuretic and non-enuretic patients with OAB (Table-1), no statistically significant difference was found between the two groups with respect to sex, age or skin color.

**Table 1:** Demographic characteristics of the enuretic and non-enuretic children.

Variable	Non-Enuretics (n)	Enuretics (n)	P-value
Male	26	58	
Female	33	83	p=0.702
Age ≤ 9 years	41	87	
Age ≥ 10 years	18	54	p=0.295
Skin color not black	14	29	
Skin color black	27	80	p=0.363
**Total**	**59**	**141**	

The clinical characteristics of both groups are showed in Table-2. The symptoms associated with the presence of NE were urgency (p=0.001), urge incontinence (p=0.001) and holding maneuvers (p=0.033). There was no difference in relation to the presence of febrile or afebrile urinary infection, nocturia, constipation, straining or abnormal urine flow. In addition, there was no statistically significant difference between those patients with severe enuresis (≥3 episodes/week) and the nonenuretic patients in relation to any of these factors. Following multivariate analysis, only one independent predictive factor was identified: the presence of holding maneuvers (p=0.022).

**Table 2:** Urinary symptoms in enuretic and non-enuretic children.

Variable	Non-Enuretics (n/%)	Enuretics (n/%)	P-value
UTI without fever	24 (42.9)	45 (36.9)	p=0.448
UTI with fever	24 (44.4)	50 (40.7)	p=0.637
**Urgency**	**48 (81.4)**	**135 (95.7)**	**p=0.001**
**Urge incontinence**	**37 (63.8)**	**120 (85.7)**	**p=0.001**
**Pollakiuria**	27 (46.6)	**83 (60.6)**	**p=0.071**
Dysfunctional urination	23 (39.0)	44 (31.2)	p=0.288
Nocturia	19 (33.3)	35 (24.8)	p=0.223
**Holding maneuvers**	**34 (59.6)**	**102 (75.0)**	**p=0.033**
Non-urge incontinence	30 (51.7)	70 (50.4)	P=0.861

**UTI** = urinary tract infection

## DISCUSSION

Few studies have dealt with the subject of non-monosymptomatic NE. Since the principal complaint of families generally concerns nocturnal enuresis, daytime complaints are often undervalued and forgotten, hampering the implementation of appropriate treatment. In some cases, parents may even be unaware of daytime symptoms, since the frantic rhythm of modern life tends to leave parents with less time to participate in the day-to-day routine of their child's life. Nocturnal enuresis causes problems with socialization, resulting in low self-esteem and stress both of the child and the family. In addition, NE has been found to be associated with behavioral alterations such as attention-deficit/hyperactivity disorder (13).

In the present sample, enuresis was shown to be a common symptom in patients with OAB, occurring in 70.5% of cases. Despite the extent to which NE distresses parents, little attention has been paid to factors potentially associated with NE in patients with LUTS. A better understanding of this relationship may help identify possible factors associated with more severe conditions. Consequently, physicians will be able to act more effectively by implementing more individualized treatments. When performing the clinical assessment of NE, physicians have frequently neglected daytime symptoms. To the best of our knowledge, this study is innovative in that an inverse analysis was used, i.e. the presence of enuresis was investigated in patients with symptoms of OAB.

In this series of patients, both the daytime urinary symptoms and enuresis were more common in girls. This finding in children with overactive bladder differs from cases of monosymptomatic NE, which tend to be more common in boys. In a study conducted with a group of 51 patients, Naseri et al. confirmed an association between enuresis and daytime incontinence, and reported twice as many girls being affected as boys (14). In the present group of 141 children with enuresis associated with OAB, the proportion of girls to boys was 1.45 to 1.

The frequency of monosymptomatic enuresis tends to decrease progressively with age until reaching a rate of 0.5 to 1% in adults. In cases of nonmonosymptomatic enuresis, however, some studies have shown that this does not occur, with the frequency of enuresis tending to remain the same in older children, possibly as a consequence of the mechanisms involved in overactive bladder (11, 12, 15). Accordingly, the present study also failed to detect any reduction in enuresis with increasing age in non-monosymptomatic enuresis.

Children and adolescents with symptoms of overactive bladder should be investigated for nocturnal enuresis, principally in the presence of holding maneuvers. The present results suggest that one of the principal mechanisms involved in non-MSE is nocturnal overactive bladder, given that the abovementioned symptoms are characteristic of this condition. The hypothesis that these children may have more difficulty waking up than non-enuretic children has to be investigated, since the involvement of assorted physio-pathological mechanisms cannot be discarded. Furthermore, the psychological impact of NE in children with OAB, and the influence of NE in rendering prognosis poorer in these patients are factors that remain to be evaluated. It is important to note that it has been found a higher rate of psychiatric disorders in children who postpone voidind with holding maneuvers (11). Further studies should be performed in order to evaluate if it is also true for patients with non-monosymptomatic enuresis.

Constipation and bowel problems are often associated with urinary tract abnormalities and 90 (46.4%) of the patients in this series had at least one complaint. Nevertheless, the groups of enuretic and non-enuretic patients were not significantly different with respect to these symptoms. None of the other clinical characteristics of the patients with lower urinary tract dysfunction were found to be associated with NE. Factors such as the presence of urinary tract infection, irrespective of the number of episodes, and nocturia were not found to be significantly associated with enuresis.

This study has some limitations. For example, the subjective nature of the symptoms may have led to interpretation errors. Moreover, the severity of enuresis was assessed based on the responses provided by parents and children rather than by analyzing the number of dry nights recorded on a chart. However, to the best of our knowledge, although the association of OAB in patients with NE has been studied extensively, the association of NE in patients with OAB has not. The results of the present study may shed more light on the actual rate of enuresis in children with OAB and on the profile of patients who are more prone to enuresis, thus opening perspectives for new studies. The impact of enuresis on both the patients with OAB and their caregivers remains to be evaluated. Nevertheless, understanding this impact is relevant since more than half of the patients present with this symptom. Furthermore, those patients with OAB who would benefit from a simultaneous treatment for enuresis should be clearly identified.

## CONCLUSION

NE and OAB are commonly associated, with NE being present in 70.5% of children with OAB. In this study on children with overactive bladder, daytime urinary symptoms and non-monosymptomatic enuresis were both more common in girls. No reduction in enuresis was found with increasing age. Therefore, as long as overactive bladder and, consequently, daytime symptoms remain untreated, there will be no improvement in nocturnal symptoms. Of the OAB symptoms, only holding maneuvers were found to be associated with enuresis as an independent predictive factor. This suggests that when these daytime symptoms are present, OAB and NE probably share the same genesis.

## ABBREVIATIONS

LUTS: = lower urinary tract symptoms
Non-MSE: = non-monosymptomatic enuresis
OAB: = overactive bladder
NE: = nocturnal enuresis
LUTD: = lower urinary tract dysfunction
